# Pocket proteins critically regulate cell cycle exit of the trabecular myocardium and the ventricular conduction system

**DOI:** 10.1242/bio.20135785

**Published:** 2013-07-31

**Authors:** David S. Park, Rose O. Tompkins, Fangyu Liu, Jie Zhang, Colin K. L. Phoon, Jiri Zavadil, Glenn I. Fishman

**Affiliations:** 1 Leon H. Charney Division of Cardiology, New York University School of Medicine, New York, NY 10016, USA; 2 Heart Rhythm Center, New York University School of Medicine, New York, NY 10016, USA; 3 Division of Pediatric Cardiology, New York University School of Medicine, New York, NY 10016, USA; 4 Department of Pathology, New York University School of Medicine, New York, NY 10016, USA; 5 NYU Genome Technology Center, New York University School of Medicine, New York, NY 10016, USA; 6 Mechanisms of Carcinogenesis Section, WHO International Agency for Research on Cancer, 69372, Lyon, France

**Keywords:** Cardiac conduction system, Cardiogenesis, Cell cycle, Chamber development, Pocket proteins, Trabecular myocardium

## Abstract

During development, the ventricular conduction system (VCS) arises from the trabecular or spongy myocardium. VCS and trabecular myocytes proliferate at a significantly slower rate than compact zone myocardial cells, establishing a transmural cell cycle gradient. The molecular determinants of VCS/trabecular myocyte cell cycle arrest are not known. Given the importance of pocket proteins (Rb, p107 and p130) in mediating G0/G1 arrest in many cell types, we examined the role of this gene family in regulating cell cycle exit of the trabecular myocardium and ventricular conduction system. Using a combinatorial knockout strategy, we found that graded loss of pocket proteins results in a spectrum of heart and lung defects. p107/p130 double knockout (dKO) hearts manifest dysregulated proliferation within the compact myocardium and trabecular bases, while the remaining trabecular region cell cycle exits normally. Consequently, dKO hearts exhibit defective cardiac compaction, septal hyperplasia and biventricular outflow tract obstruction, while the VCS appears relatively normal. Loss of all three pocket proteins (3KO) is necessary to completely disrupt the transmural cell cycle gradient. 3KO hearts exhibit massive overgrowth of the trabecular myocardium and ventricular conduction system, which leads to fetal heart failure and death. Hearts carrying a single pocket protein allele are able to maintain the transmural cell cycle gradient. These results demonstrate the exquisite sensitivity of trabecular and conduction myocytes to pocket protein function during ventricular chamber development.

## Introduction

The ventricular conduction system (VCS), comprised of the His bundle, bundle branches, and the Purkinje fiber network, develops from the trabecular or spongy myocardium. VCS differentiation from trabecular myocytes occurs through a complex interaction of cell autonomous and paracrine signals from the closely apposed endocardium. Upon specification, the proliferative capacity of VCS/trabecular myocytes is significantly attenuated while the compact myocardial zone proliferates at a significantly faster rate, creating a transmural cell cycle gradient (Mikawa et al., 1992a; Mikawa et al., 1992b; Soonpaa et al., 1996; Meilhac et al., 2003). Differential proliferation rates between the trabecular and compact myocardial zones are essential for proper chamber development.

During chamber formation, the ventricles are committed to eccentric remodeling (or ‘chamber ballooning’) to accommodate the increasing cardiac output demands of the growing embryo. The compact myocardial zone remains relatively thin, proliferates rapidly, and maintains an immature cardiomyocyte phenotype. In contrast, trabecular myocytes cell cycle arrest early and exhibit a more mature sarcomeric structure. Therefore, the trabecular myocardium provides most of the contractile force during the ‘ballooning’ phase of ventricular development. Early specification of the VCS within the trabecular region allows for rapid impulse propagation throughout the spongy myocardium optimizing synchronized contraction of the ventricles. Indicative of their early cell cycle withdrawal, cells of the cardiac conduction system comprise less than 1% of the adult cardiomyocyte population. Although significant advances have been made in unraveling the transcription factor pathways that regulate trabecular and conduction system development, very little is known about the cell cycle machinery governing this process (Shou et al., 1998; Chen et al., 2004; Pashmforoush et al., 2004; Grego-Bessa et al., 2007; Bakker et al., 2008).

Cell cycle progression is a highly regulated process that depends on transcriptional regulation, ubiquitin-mediated degradation, and post-translational modification of cell cycling proteins. Mitogenic signals are transduced into cyclically expressed proteins known as cyclins that when coupled with cyclin-dependent kinases (CDK) orchestrate the passage through regulatory checkpoints (Sherr, 1994). Cyclin–CDK complexes phosphorylate members of the pocket protein gene family (*Retinoblastoma (Rb), p107, p130*) resulting in their inactivation (Ewen et al., 1992; Faha et al., 1992; Hannon et al., 1993; Li et al., 1993).The pocket proteins represent one of the final common pathways in the cell cycle regulatory cascade and have been implicated in apoptosis, DNA damage repair, differentiation, and development (Jacks et al., 1992; Lee et al., 1996; Cobrinik, 2005).

Pocket proteins mediate G0/G1 arrest in numerous cell types, in part through repression of E2F transcription factors (Dimova and Dyson, 2005). E2Fs regulate the expression of genes involved in G1/S and G2/M transitions. Hypo-phosphorylated (active) pocket proteins inhibit S phase entry via interaction with the E2F transactivation domain (Zhu et al., 1995a; Zhu et al., 1995b). Hypo-phosphorylated Rb binds both repressive (E2F4) as well as activating E2F's (E2F1, 2, 3A/B), inhibiting their transcriptional activity (Lees et al., 1993; Dimova and Dyson, 2005). Hypo-phosphorylated p107 and p130 bind to repressive E2Fs (E2F4 and E2F5) and the resultant complexes repress E2F-responsive promoters through recruitment of histone deacetylases (Ginsberg et al., 1994; Hijmans et al., 1995; Dimova and Dyson, 2005). Phosphorylation of pocket proteins via activated cyclin–CDK complexes results in release of E2F transcription factors, allowing for passage through cell cycle checkpoints (Beijersbergen et al., 1995; Dimova and Dyson, 2005).

Pocket proteins are important in maintaining adult cardiomyocyte quiescence, but there remains no conclusive evidence that Rb family members regulate cell cycle arrest of fetal cardiomyocytes or of specialized conduction cells. p107 and p130 are highly expressed in the developing heart, while cardiac Rb expression is restricted to late fetal and post-natal stages (Jiang et al., 1997; MacLellan et al., 2005). Despite this enrichment of p107 and p130 during development, a cardiac phenotype was not reported in p107/p130 double knockout mice (Cobrinik et al., 1996). Combinatorial knockout of cardiac Rb and p130 resulted in proliferative defects in adult cardiomyocytes, but neonatal hearts were normal (MacLellan et al., 2005). Whether adult conduction myocytes were equally affected in this model was not explored. Embryo-restricted loss of Rb in a p107 null background resulted in double outlet right ventricle due to dysregulated proliferation of endothelial and possibly heart mesenchymal cells, but again cardiomyocyte proliferation was normal (Berman et al., 2009). Taken together, these results reinforced the notion that pocket proteins have little to no role in cardiomyocyte or VCS cell cycle arrest during development. Furthermore, the recent identification of pocket protein-independent pathways regulating G0/G1 arrest in teratoma and cell culture models underscores the need to validate pocket protein networks in specific cell types *in vivo* (Wirt et al., 2010).

In this report, we investigate the role of pocket proteins in mediating cell cycle arrest of the developing ventricular conduction system and trabecular myocardium. We demonstrate that all three pocket protein family members are expressed in the VCS/trabecular myocardium. Using a combinatorial knockout strategy, we show that graded loss of pocket proteins results in a spectrum of heart and lung defects. While p107/p130 dKO mice exhibit enhanced proliferation within zones of active cell cycling (compact myocardium and trabecular bases), triple knockout embryos manifest full disruption of the transmural cell cycle gradient. Complete disruption of trabecular myocyte cell cycle control results in massive overgrowth of the ventricular conduction system leading to outflow tract obstruction, impaired ventricular filling, heart failure, and death. These results unequivocally demonstrate the critical importance of pocket proteins in maintaining trabecular and VCS cell cycle control during ventricular chamber development.

## Results

### The trabecular myocardium is predominantly in G0/G1 phase of the cell cycle throughout development

**Fig. 1.**
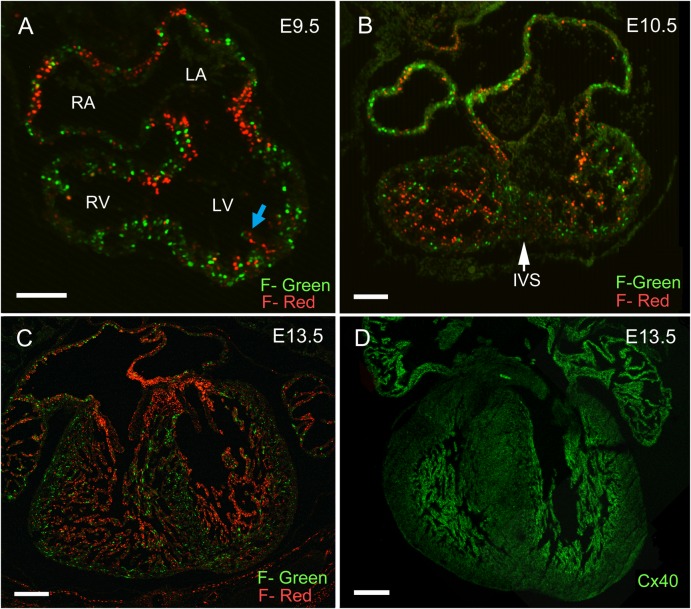
Cell cycle characterization of the developing heart using the FUCCI transgenic mouse. FUCCI-Red and Green double transgenic embryos were studied at E9.5 (**A**), E10.5 (**B**), and E13.5 (**C**). (A) At E9.5 the heart had undergone D-looping and AV canal myocytes were arrested in G0/G1 phase creating a narrow waist. Trabecular myocytes were seen budding from the subendocardial surface and were arrested in G0/G1 phase (blue arrow), while cells of the compact region continued to cell cycle. (B) At E10.5, the trabecular region had markedly expanded although the majority of cells were in G0/G1 phase. An increased proportion of trabecular basal myocytes located at the interface with the compact myocardium were in S/G2/M phases. (C,**D**) At E13.5, development of the trabecular myocardium was complete and FUCCI-Red trabecular cells overlapped with Connexin 40 (Cx40) expression. (FUCCI-Green, F-Green; FUCCI-Red, F-Red). Scale bars: 20 µm (A,B), 200 µm (C,D).

Cell cycling characteristics of the developing heart were analyzed using the FUCCI mouse system, which allows direct visualization of cell cycle phases *in vivo* (Sakaue-Sawano et al., 2008). Using anti-phase, oscillating proteins (Cdt and Geminin) tagged with fluorescent markers, cells in G0/G1 phases label red, while cells in S/G2/M phases label green. Cell cycle analysis of E12.5 dissociated cardiomyocytes from double FUCCI-Red and -Green transgenic embryos confirmed the validity of this model in the developing heart (supplementary material Fig. S1). As shown in Fig. 1, FUCCI-Red/Green embryos were harvested at different embryonic timepoints and the cell cycling characteristics of the fetal heart were defined. At E9.5, the D-looped heart initiates chamber specification, which is characterized by ‘chamber ballooning’ and trabecular formation along the endocardial surface (Fig. 1A). Cardiomyocytes in the outer curvature have a high proportion of cells in S/G2/M phases as evidenced by the abundance of green nuclei. This is in contrast to trabecular myocytes, which are predominantly in G0/G1 phase soon after they bud from the underlying primitive myocardium (Fig. 1A, blue arrow). Myocytes within the atrioventricular (AV) canal are arrested in G0/G1 phase giving rise to the narrow waist of the AV ring (Fig. 1A).

At E10.5, the interventricular septum begins as a protuberance of proliferating myocytes on the dorsal aspect of the interventricular groove (Fig. 1B, arrow). The trabecular myocardium rapidly expands to become the predominant myocyte in the ventricular chambers, with the vast majority of trabecular cells in G0/G1 phase (Fig. 1B). Notably, the trabecular myocytes at the interface with the compact myocardial layer have a higher percentage of cells in S/G2/M phase, consistent with the growth plate being localized to the base of trabeculae (Fig. 1B). At E13.5, cardiac septation takes place with closure of the endocardial cushions and formation of the four-chambered heart. At this time, trabecular development is complete, and the distribution of G0/G1 arrested cells overlaps with Connexin40 (Cx40) expression within the trabecular region and the developing ventricular conduction system (Fig. 1C,D). Subsequently, the trabecular myocardium coalesces to form trabeculae carnae, papillary muscles, and the ventricular conduction system.

### p107 and p130 protein expression colocalize in the developing trabecular region

**Fig. 2.**
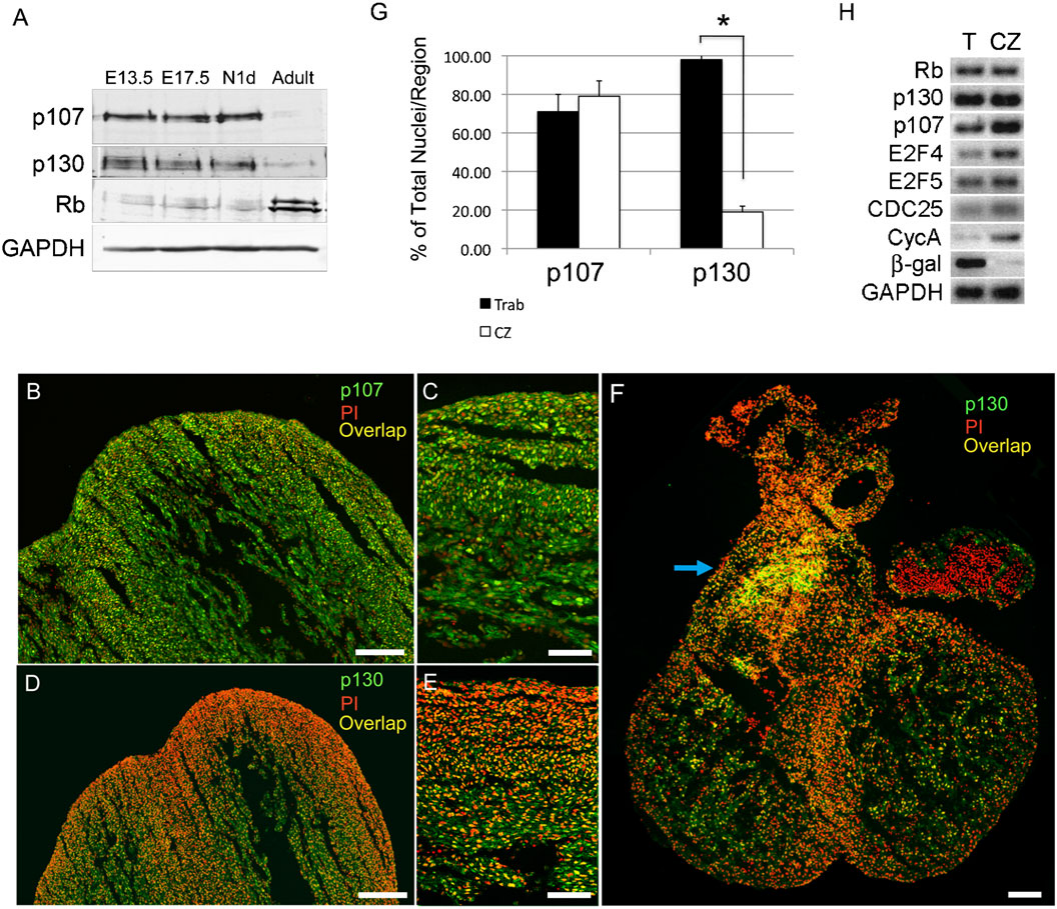
Pocket protein expression was evaluated temporally and spatially during cardiac development. (**A**) p107 and p130 were the dominant pocket proteins expressed in the heart during the embryonic and neonatal period. Rb was expressed in a reciprocal pattern with p107 and p130 with highest expression in the adult heart. (**B–F**) The regional expression of p107 and p130 was examined in E12.5 wild type hearts. While the expression of p107 appeared uniformly distributed throughout the trabecular and compact myocardium (B,C), p130 expression was enriched in the trabecular region (D,E). (F) The expression of p130 extended into the trabeculated region of the RV outflow tract (blue arrow). (**G**) The percentage of p107 and p130 positive nuclei within the trabecular and compact myocardial regions was quantified. The percentage of p107 positive nuclei was slightly lower in the trabecular region, but the difference did not reach statistical signficance (71%±9% vs 79%±8%; *P* = 0.28). The percentage of p130 positive nuclei, on the other hand, was significantly higher in the trabecular region compared to the compact zone (98%±5% vs 19%±3%; *P*<2×10^−7^). (**H**) Laser capture microdissection and differential gene expression within the trabecular versus compact myocardium. (Trabecular, T or Trab; Compact Zone, CZ; Propidium Iodide, PI). Scale bars: 100 µm (B,D,F), 50 µm (C,E).

The temporal–spatial expression of pocket proteins was evaluated during murine heart development (Fig. 2). Cardiac expression of p107 and p130 was robust during embryogenesis and into the neonatal period, whereas Rb expression was detectable at low levels in the developing heart. In the adult heart the reciprocal was true where Rb was the dominant pocket protein expressed, while p107 and p130 expression was significantly down regulated (Fig. 2A). The regional distribution of p107 and p130 protein was next evaluated in E12.5 wild type hearts using immunofluorescence staining. (Fig. 2B–F). The pattern of p107 distribution appeared similar between the compact and trabecular regions (Fig. 2B,C), while p130 expression was highly enriched in the trabecular myocardium (Fig. 2D,E). The expression of p130 extended into the trabecular region of the developing right ventricular outflow tract (Fig. 2F, blue arrow). The percentage of p107 and p130 positive nuclei was quantified within the trabecular and compact myocardial regions (Fig. 2G). The overall percentage of p107 positive nuclei was slightly lower in the trabecular region than in the compact zone, although this difference did not reach statistical significance (trabecular vs compact zone; 71%±9% vs 79%±8%; *P* = 0.28). On the other hand, the percentage of p130 positive nuclei was significantly higher in the trabecular region compared to the compact zone (trabecular vs compact zone; 98%±5% vs 19%±3%; *P*<2×10^−7^) (Fig. 2G).

Using laser capture microdissection, trabecular versus compact zone myocytes were isolated from E12.5 CCS-LacZ (an established marker of the cardiac conduction system) hearts and subjected to RT-PCR analysis (Fig. 2H) (Rentschler et al., 2001). β-galactosidase transcript was only detectable in the trabecular/VCS zone demonstrating the specificity of the microdissection. Both Cyclin A and CDC25, markers of S phase and M phase transcriptional activity, respectively, were higher in the compact region, consistent with the FUCCI analysis (Fig. 2H). p107 transcript levels were higher in compact zone myocytes, while Rb and p130 RNA levels were uniformly expressed in both myocardial regions (Fig. 2H). These data are consistent with known p107 mRNA accumulation during S/G2/M phases, whereas Rb and p130 transcript levels stay relatively constant throughout the cell cycle (Graña et al., 1998). Unlike Rb and p107, p130 protein expression is post-transcriptionally regulated (Graña et al., 1998).

### Individual pocket protein deficient mice do not exhibit heart defects or cell cycling abnormalities

**Fig. 3.**
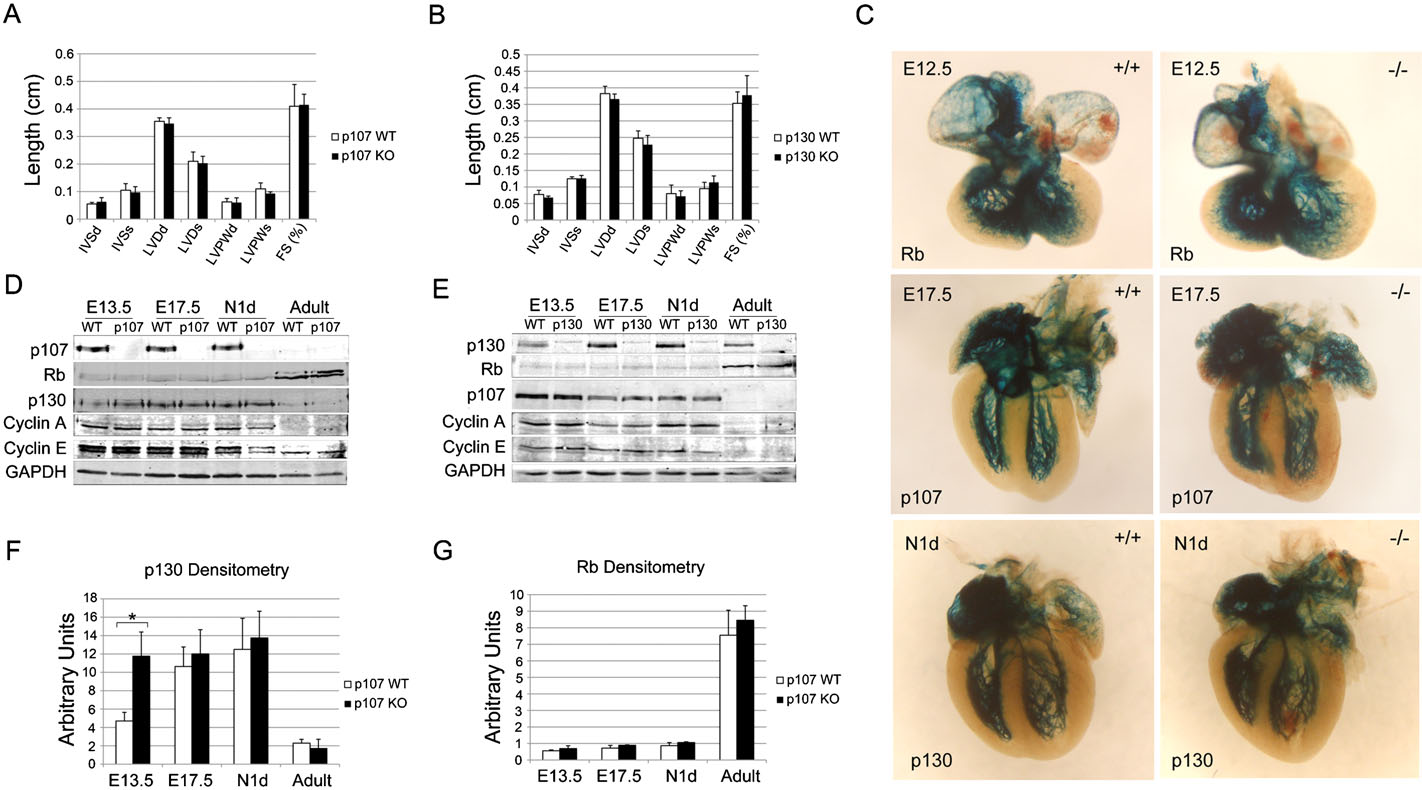
Characterization of individual pocket protein deficient mice. Transthoracic echocardiography was performed on individual p107^−/−^ (**A**) and p130^−/−^ (**B**) mice; neither displayed structural or functional abnormalities. (**C**) Patterning of the cardiac conduction system was normal in p107 and p130 single knockout mice back-crossed into CCS-LacZ reporter lines. The expression of cell cycling proteins was evaluated in p107^−/−^ (**D**) and p130^−/−^ (**E**) ventricles. (**F**) p107^−/−^ ventricles consistently showed compensatory upregulation of p130 expression at the E13.5 time point. (**G**) Rb expression did not change significantly in p107^−/−^ ventricles. (Post-natal day 1, N1d; Cyclin A, CycA; Interventricular septum during diastole and systole, IVSd and IVSs; Left ventricular dimension during diastole and systole, LVDd and LVDs; Left ventricular posterior wall thickness during diastole and systole, LVPWd and LVPWs; Fractional shortening, FS) (see also supplementary material Table S1).

Based on the robust expression of p107 and p130 in the developing heart and their redundant expression in the trabecular myocardium, we evaluated pocket protein knockout mouse lines for both structural and functional cardiac defects. Individual p107 and p130 knockout mice are grossly normal and have normal life spans, as previously reported (Cobrinik et al., 1996; Lee et al., 1996). Normal cardiac structural and functional indexes were noted for both p107 and p130 single knockout mice as measured by transthoracic echocardiography and electrocardiography (EKG) (Fig. 3A,B; supplementary material Table S1). To evaluate for patterning abnormalities of the ventricular conduction system, *Rb*, *p107* and *p130* individual knockout mice were backcrossed into CCS-LacZ reporter mice. No discernible patterning defects were noted in the His bundle, bundle branches, or Purkinje fiber network of individual pocket protein knockout hearts (Fig. 3C).

Cell cycling characteristics of individual p107 and p130 knockout hearts were then evaluated in a FUCCI background. The trabecular myocardium of p107 and p130 single knockout hearts retained the ability to arrest in G1 phase compared to littermate controls (data not shown). We next evaluated the protein levels of pocket proteins and cyclins A and E in individual p107^−/−^ and p130^−/−^ ventricles to assess for compensation and/or perturbation of G1 and S phase cyclin expression (Fig. 3D,E). Consistent with the FUCCI analysis, no significant change in cyclin A or E expression was noted in individual pocket protein knockout hearts. However, protein levels of p130 were significantly up regulated in *p107^−/−^* hearts at E13.5, a key time point marking the end of trabecular development and the beginning of cardiac compaction (Fig. 3F). Rb protein levels remained unchanged at all developmental timepoints in individual p107^−/−^ and p130^−/−^ hearts (Fig. 3G).

### *p107^−/−^/p130^−/−^* double knockout (dKO) mice are cyanotic at birth and exhibit neonatal lethality from severe lung defects

**Fig. 4.**
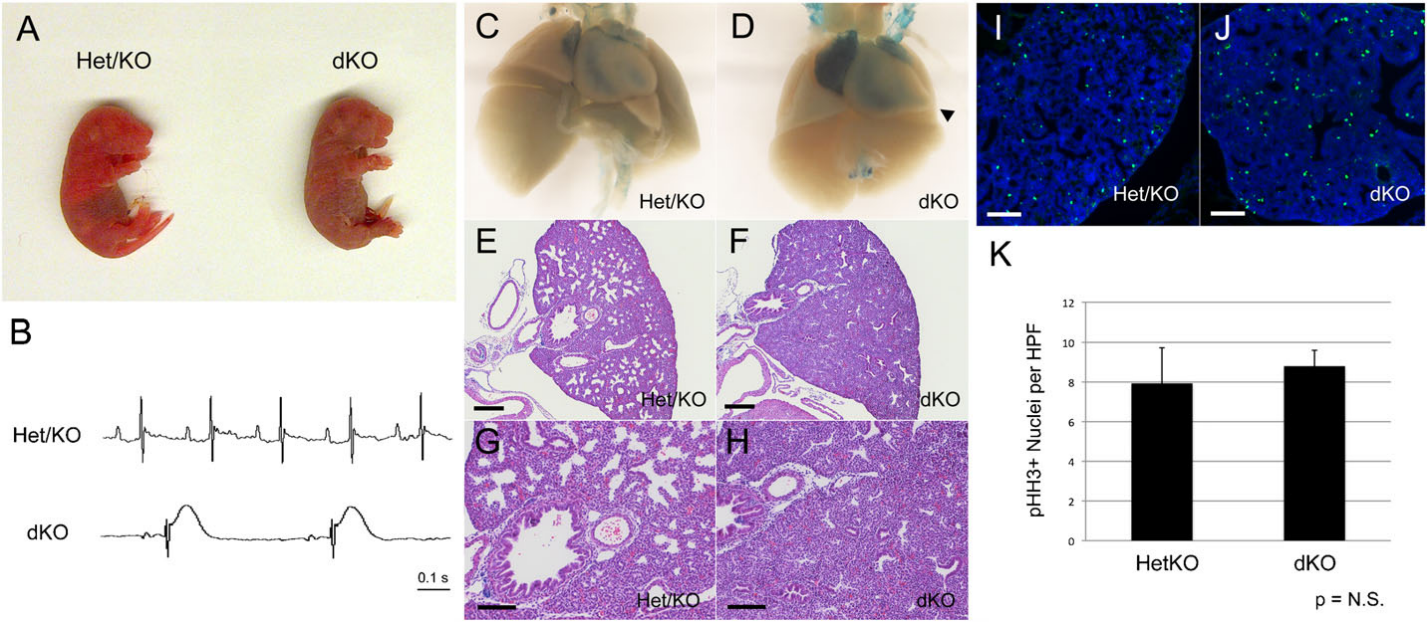
Lung defects in dKO mice. (**A**) dKO pups were cyanotic at birth. (**B**) The electrocardiograms of dKO pups exhibited marked sinus bradycardia with ST segment elevation. (**C**,**D**) dKO/CCS-LacZ heart and lungs at E17.5 were removed intact and X-gal stained. dKO ventricular chambers were enlarged with LV apical protrusion noted in all dKO hearts (arrowhead). dKO lungs appeared grossly normal morphologically but were slightly diminished in size and had reduced translucency. (**E–H**) dKO lungs had impaired alveolar development giving the lungs the appearance of hypercellularity. (**I–K**) Quantitative phospho-Histone H3 (pHH3) staining did not show a significant increase in the number of mitotic cells in dKO lungs (Ctl vs dKO; 7.9±1.8 vs 8.8±0.8; *P* = 0.48). Scale bars: 200 µm (E,F), 100 µm (G–J).

The significant increase in p130 protein levels in p107^−/−^ ventricles at E13.5 raised the possibility that compensation was playing a role in mitigating loss of cell cycle control in the trabecular myocardium. To investigate this possibility, we generated *p107^−/−^/p130^−/−^* double knockout (dKO) mice and analyzed them for cardiac defects. Mice deficient in both p107 and p130 die hours after birth with visible cyanosis, as previously described (Fig. 4A) (Cobrinik et al., 1996). Electrocardiograms performed on newborn dKO pups revealed severe bradycardia and marked ST segment abnormalities consistent with myocardial ischemia (Fig. 4B). As marked cyanosis can be due to pulmonary disease or congenital heart disease, dKO mice were evaluated histologically for cardiopulmonary abnormalities. dKO mice exhibited significant developmental abnormalities in both the lung and the heart. Newborn litters with cyanotic pups were sacrificed and the hearts and lungs removed intact. The heart/lung samples of non-cyanotic littermates were buoyant when placed in saline solution. In contrast, dKO heart/lungs had diminished buoyancy or sank suggestive of poor lung aeration. Gross examination of E17.5 dKO lungs revealed a slight reduction in lobar size and translucency (Fig. 4D). dKO/CCS-LacZ hearts stained with X-gal consistently demonstrated RV enlargement and marked LV apical protrusion (Fig. 4D, arrowhead). Histological evaluation of dKO lungs revealed severely impaired alveolar development when compared to *p107^+/−^/p130^−/−^* (Het/KO) littermates (Fig. 4E–H). Quantitative analysis of phospho-Histone H3 positive nuclei (established marker of mitosis) did not reveal a significant increase in mitotic cells in E14.5 dKO lungs (Fig. 4I–K). Therefore, the hypoxic phenotype in dKO pups is due to abnormal alveolar development, which does not appear to be a consequence of cell cycle dysregulation in the fetal lungs, at least at the stage analyzed.

### DKO hearts are grossly elongated and display infundibular pulmonary stenosis and left ventricular outflow tract (LVOT) obstruction

**Fig. 5.**
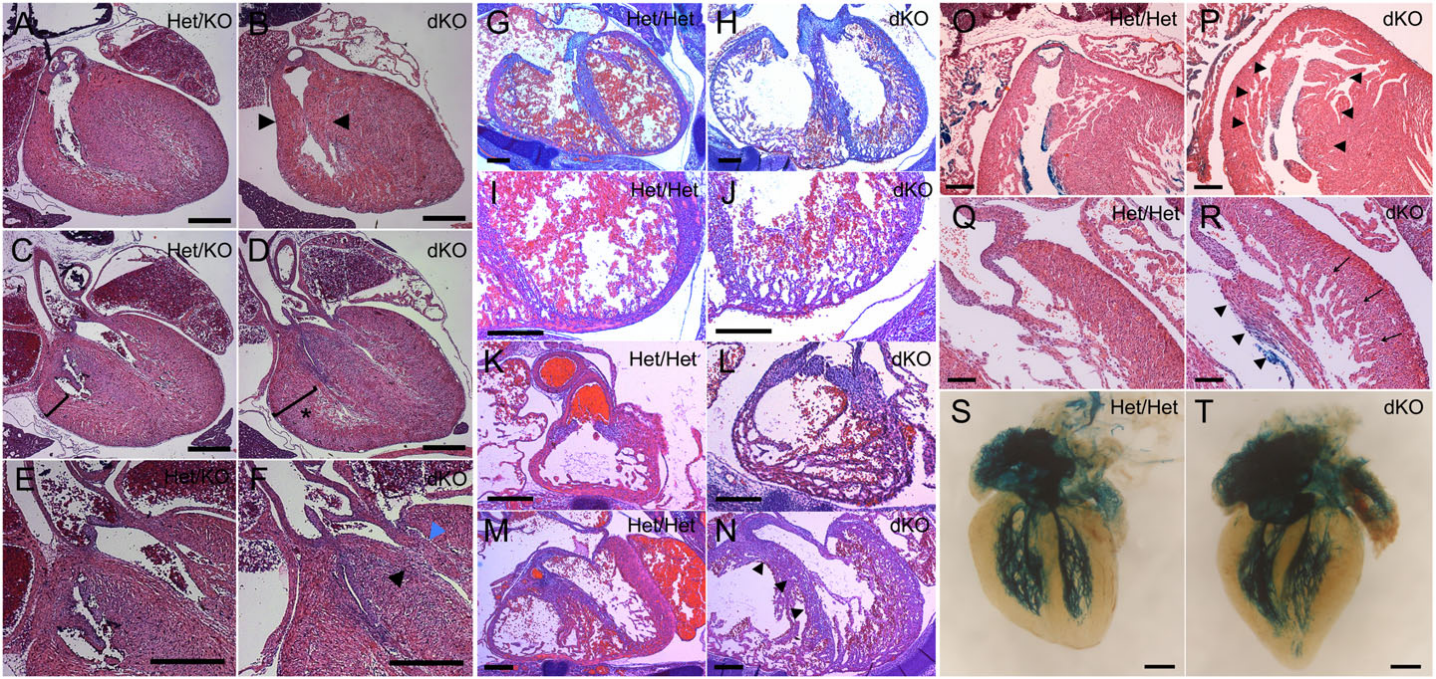
Cardiac defects in dKO embryonic hearts at N1d (A–F), E14.5 (G–N), and E17.5 (O–T). (A–F) dKO hearts exhibited a constellation of defects, which included: (B) thickening of the septomarginal and parietal trabeculations (arrowheads) leading to infundibular pulmonary stenosis, (D) RV hypertrophy was evident (asterisk), and (F) LVOT obstruction was noted due to displacement of the septal crest into outflow tract (black arrowhead) and thickening of the anterior papillary muscle (blue arrowhead). (G–N) E14.5 dKO hearts showed defective cardiac compaction and eccentric remodeling in the IVS (H), LV (J), RVOT (L) and LVOT (N). (N) Marked elongation of the IVS in dKO hearts caused redundancy and encroachment of the septum into the LVOT (arrowheads). (O,P) At E17.5, severe thickening of the septo-marginal and parietal trabeculations resulted in narrowing of the RVOT (P, arrowheads). (R) Persistently delayed cardiac compaction in E17.5 dKO hearts was evident as prominent trabeculations (arrows) and thickened papillary muscles (arrowheads). (T) dKO hearts showed marked elongation of the ventricles and IVS resulting in abnormal twisting of the LV with apical protrusion. (T) The overall patterning of the ventricular conduction system was normal in dKO hearts. Scale bars: 500 µm (A–F), 200 µm (G–P), 100 µm (Q,R), 300 µm (S,T).

We next evaluated dKO newborn pups for cardiac abnormalities. Postnatal day 1 (N1d) dKO hearts demonstrated bi-ventricular outflow tract obstruction, IVS elongation with over-riding aorta, and right ventricular hypertrophy (Fig. 5B,D,F). In the right ventricular outflow tract (RVOT), marked thickening of the septo-marginal and parietal trabeculae resulted in variable degrees of infundibular pulmonic stenosis (IPS) (Fig. 5B, arrowheads). Significant right ventricular hypertrophy (RVH) was also seen (Fig. 5D, asterisk). As IPS may be a secondary response to pulmonary valvular stenosis, detailed inspection of the pulmonary valve was performed, which revealed no evidence of pathology (data not shown). Marked elongation of the interventricular septum (IVS) resulted in displacement of the septal crest into the left ventricular outflow tract (LVOT) creating obstruction and an over-riding aorta (Fig. 5F, arrowheads). Thickening of the anterior papillary muscle of the mitral valve further exacerbated LVOT obstruction (Fig. 5F, blue arrowhead).

### Hearts of dKO pups exhibit cell cycling abnormalities and defective cardiac compaction

Histological analysis of E14.5 dKO hearts revealed defective cardiac compaction throughout all ventricular myocardial regions compared to Het/Het littermate controls (Fig. 5G–N). Delayed compaction was associated with significant LV and RV eccentric remodeling (Fig. 5H,J,L,N) and marked elongation of the interventricular septum (Fig. 5N, arrowheads). dKO E14.5 ventricles did not appropriately switch from eccentric remodeling to concentric remodeling, and thus maintained a more immature ventricular morphology. At E17.5, the dKO right ventricle was hypertrabeculated with severe thickening of the septo-marginal and parietal trabeculations causing narrowing of the RVOT (Fig. 5P, arrowheads). In the dKO left ventricle, cardiac compaction, which was now evident, was accompanied by prominent trabeculations with thickening and elongation of the papillary muscles (Fig. 5R, arrows and arrowheads, respectively). Prominent trabeculations were matched by significant elongation of the ventricles and IVS, creating an abnormal twisting of the LV with apical protrusion (Fig. 5T). Despite these structural changes, patterning of the ventricular conduction system was relatively normal in dKO hearts (Fig. 5T).

**Fig. 6.**
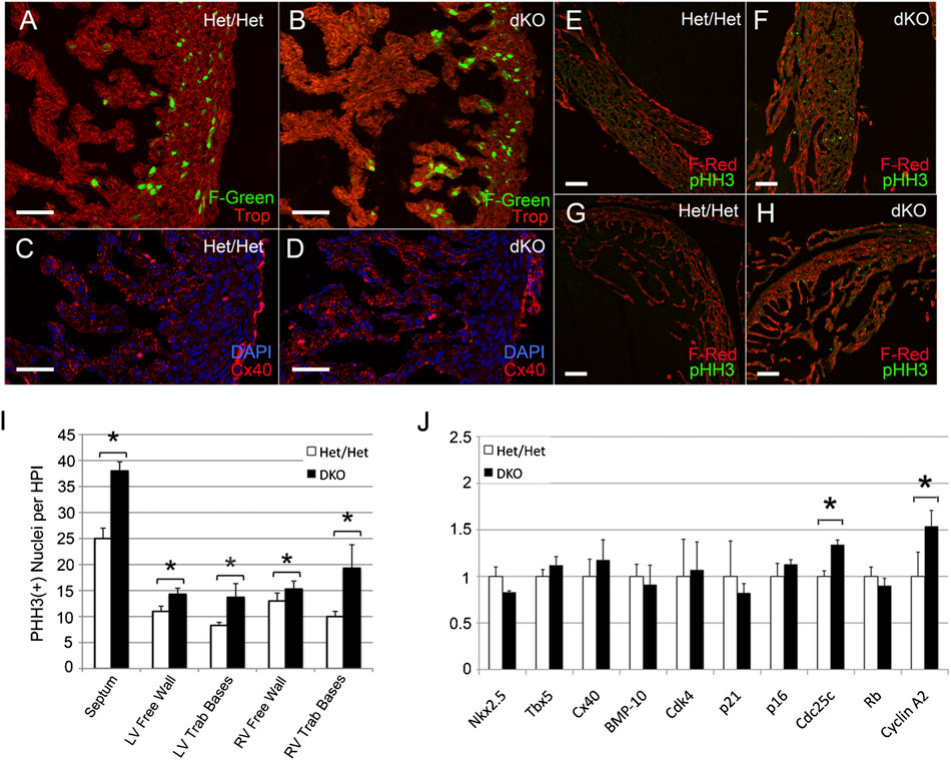
Analysis of dKO/Fucci-Green (dKO/F-Green) and dKO/Fucci-Red (dKO/F-Red) hearts (A–H). (B) Non-compacted E14.5 dKO/F-Green hearts remained committed to trabecular expansion and eccentric remodeling as evidenced by persistent F-Green positive nuclei at trabecular bases. (D) The hypertrabecularized region was composed of Cx40 positive myocytes. (E–H) dKO/F-Red hearts at E15.5 were immunostained for pHH3 to quantify the number of cells undergoing mitosis. (F,H) dKO hearts exhibited a significant increase in mitotic cells throughout the compact myocardium and in the trabecular bases. (**I**) The number of mitotic cells in the interventricular septum, LV and RV compact myocardium, and trabecular bases was quantified per high power field (*P<0.05). (**J**) Semi-quantitative RT-PCR analysis of E14.5 dKO ventricles vs Het/Het littermate controls. Both Cyclin A and CDC25 were significantly higher in dKO ventricles (*P<0.05). Scale bars: 50 µm (A–D), 100 µm (E–H).

Analysis of E14.5 *p107^+/−^/p130^+/−^*/FUCCI-Green (Het/Het/F-Green) hearts showed stage-appropriate enrichment of F-Green nuclei in the expanding compact myocardial zone, while only rare F-Green nuclei were present in the trabecular compartment (Fig. 6A). In contrast, dKO/F-Green hearts remained committed to trabecular expansion and eccentric remodeling, as evidenced by persistent F-Green positive nuclei at the base of trabeculae and in the thin compact myocardial zone (Fig. 6B). The number of F-Green positive nuclei in the remaining trabecular regions was similar to the levels seen in Het/Het littermate controls. Cx40 staining confirmed that the hyper-trabecularized region was composed of trabecular myocytes, as opposed to non-compacted free wall myocytes (Fig. 6C,D).

dKO;F-Red hearts at E15.5 were immunostained for phospho-histone H3 to quantify the number of cells undergoing mitosis (Fig. 6E–H). dKO hearts exhibited a significant increase in mitotic cells throughout the compact myocardium and in the trabecular bases (Fig. 6F,H). The number of mitotic cells in the septum and compact myocardium increased by approximately 1.5- and 1.2-fold, respectively, and in the LV and RV trabecular bases the number of mitotic cells increased by approximately 1.6- and 2-fold, respectively (Fig. 6I). Expression of both Cyclin A and Cdc25 were significantly increased in E14.5 dKO ventricles compared to Het/Het littermate controls (Fig. 6J). There was no significant change in the expression of Rb or of trabecular differentiation markers. These results suggest that the degree of hypertrabeculation is matched by a proportional increase in compact myocardial components.

### Pocket protein 3KO hearts have profound cell cycling abnormalities in trabecular/VCS cardiomyocytes

**Fig. 7.**
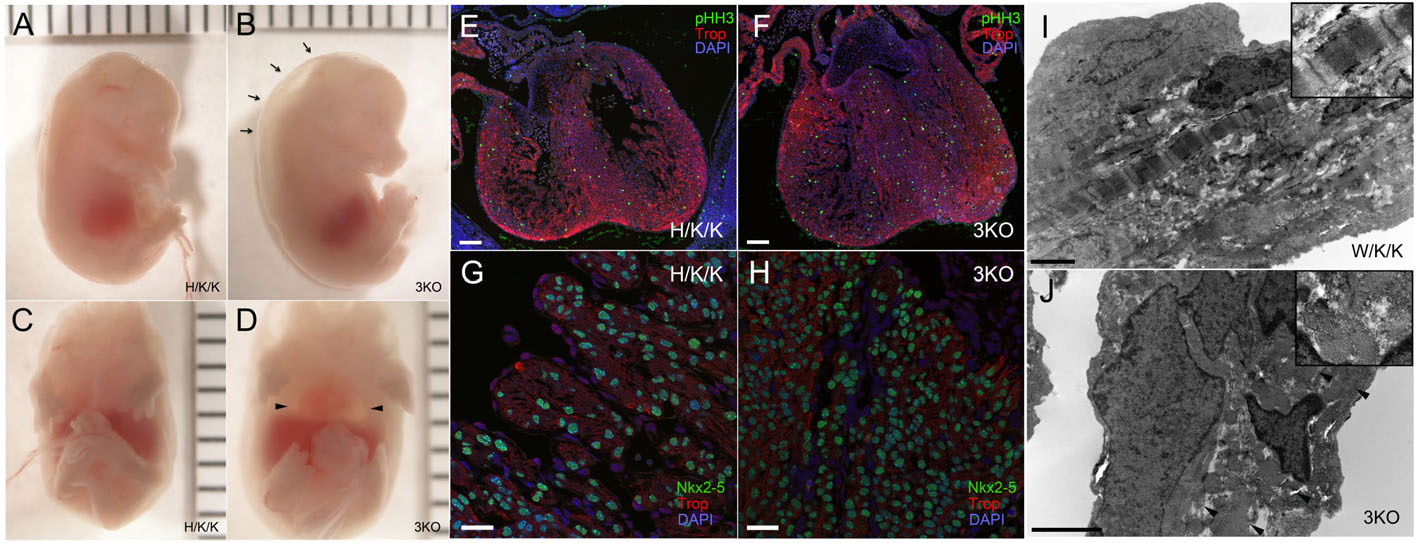
Characterization of 3KO embryos at E15.5. (**A–D**) A subset of E15.5 3KO pups showed evidence of heart failure with peripheral edema (B, arrows) and large pericardial effusions (D, arrowheads). (**E–H**) 3KO embryos and H/K/K littermates were subjected to immunofluorescent staining. (F) 3KO hearts at E15.5 had complete disruption of the transmural cell cycle gradient. Ventricular chambers were filled with troponin positive trabecular myocytes with an equalized distribution of pHH3 positive cells throughout the ventricular wall. (H) The overgrown trabecular region was filled with Nkx2-5 positive nuclei consistent with loss of cardiomyocyte cell cycle control. Compared to H/K/K littermate controls (G), 3KO nuclear to cytoplasmic ratios were significantly reduced (H). (**I**) Trabeculae of W/K/K littermates were developmentally advanced with mature sarcomeric structure (inset). (**J**) In contrast, 3KO trabeculae were notable for numerous mitotic figures and sarcomeres that were in various stages of disassembly/reassembly (arrowheads and inset). Scale bars: 1 mm (A–D), 100 µm (E,F), 25 µm (G,H), 2 µm (I,J).

Despite abnormalities of cardiac compaction and cell cycling in dKO hearts, the transmural cell cycle gradient was preserved. To test whether residual Rb expression is sufficient to maintain trabecular myocyte cell cycle arrest, we generated cardiomyocyte-restricted pocket protein-deficient mice (p107^−/−^/p130^−/−^/Rb^Lox/Lox^/α-MHC-Cre^+/−^ or 3KO). As opposed to p107^+/−^/p130^−/−^/Rb^Lox/Lox^/α-MHC-Cre^+/−^ (Het^p107^/KO^p130^/KO^αMHC-Rb^, H/K/K) and p107^−/−^/p130^+/−^/Rb^Lox/Lox^/α-MHC-Cre^+/−^ (KO^p107^/Het^p130^/KO^αMHC-Rb^, K/H/K) mice, which survive to adulthood, 3KO pups were stillborn or died at E14.5–E15.5 from heart failure as manifested by large pericardial effusions and anasarca. (Fig. 7B,D). Severely affected embryos exhibited minimal ventricular filling and contraction. On histological evaluation, 3KO ventricular chambers were filled with sheets of trabecular myocytes (Fig. 7F). Double staining with pHH3 and troponin showed marked upregulation of mitotic myocytes throughout the heart with complete disruption of the normal transmural cell cycle gradient (Fig. 7E,F). The frequency of pHH3 positive cells was equally distributed between the compact and trabecular zones (Fig. 7F). Failure to cell cycle arrest resulted in crowding of Nkx2-5 positive nuclei within the trabecular region with reduced cytoplasmic to nuclear ratios, giving the appearance of immature cardiomyocytes typically restricted to the compact myocardial zones (Fig. 7H). Electron microscopy of W/K/K E14.5 trabecular myocytes demonstrated rare mitotic figures and well-organized sarcomeric structure (Fig. 7I, inset). In contrast, 3KO trabeculae were notable for numerous mitotic figures and sarcomeres that were in various stages of disassembly/reassembly (Fig. 7J, arrowheads and inset).

**Fig. 8.**
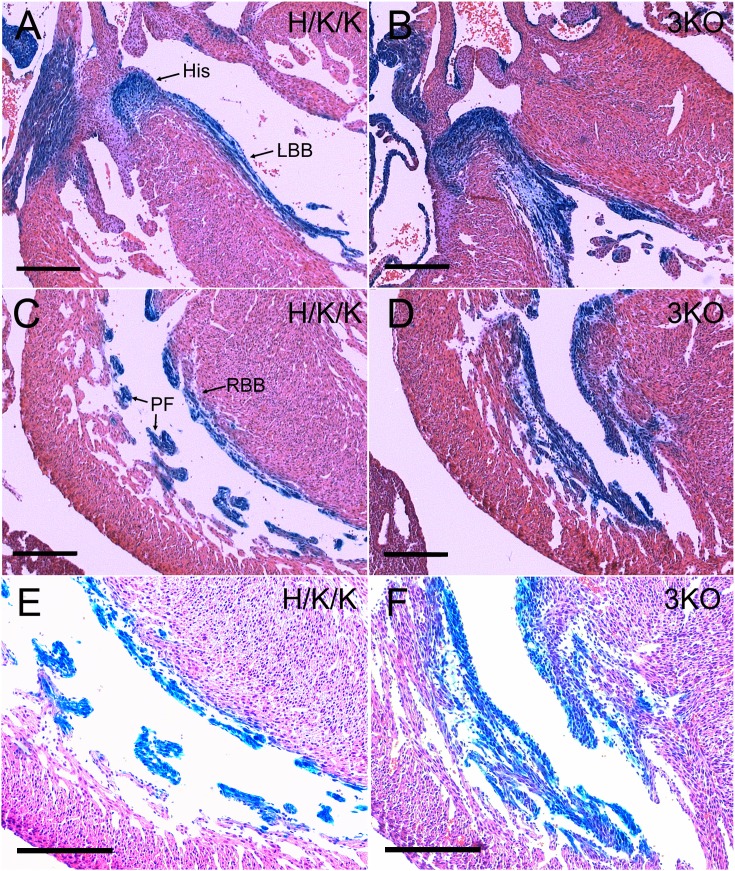
The cohort of 3KO/CCS-LacZ embryos that survived to later timepoints showed marked hyperplasia of the ventricular conduction system (A–F). (B) Severe enlargement of the His bundle and left bundle branch resulted in LVOT obstruction. (D) The right bundle branch and Purkinje network were markedly hyperplastic and thickened. (F) A higher magnification and over-exposure of the right bundle branch and Purkinje fibers demonstrated equilibration of nuclear densities between the VCS/trabecular myocytes and the compact myocardium. Scale bars: 200 µm.

**Fig. 9.**
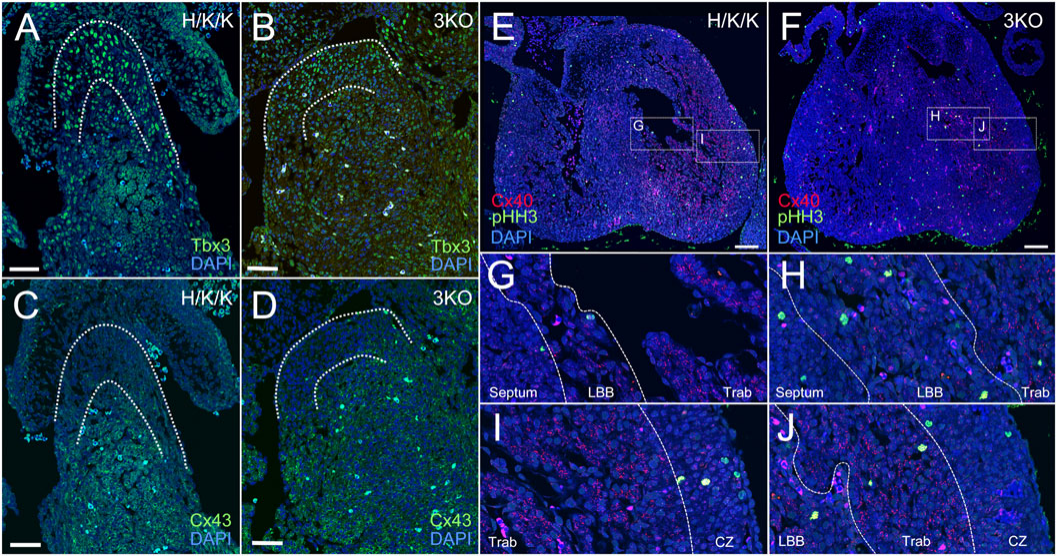
3KO hearts maintain appropriate localization of proximal VCS and trabecular markers. (**A**,**C**) In H/K/K littermates, Tbx3 was localized to the proximal VCS, where it repressed Cx43 expression. (**B**,**D**) The reciprocal pattern of Tbx3 and Cx43 expression was maintained in the 3KO proximal VCS. (**E**,**F**) In comparison to littermate hearts, 3KO ventricular chambers were filled with two populations of Cx40 positive myocytes. (**G**) The left bundle branch (LBB) in control hearts was identified as a thin band of Cx40 low-expressing myocytes adjacent to the septum. (G,**I**) The remaining trabecular myocardium (Trab) was composed of Cx40 high-expressing myocytes. (**H**,**J**) 3KO ventricles showed marked expansion of Cx40 low expressing, left bundle branch (LBB) myocytes and lateralization of Cx40 high-expressing trabecular myocytes (Trab) within the LV chamber. (I,J) The boundary between the trabecular region (Trab) and the compact myocardium (CZ) remains intact in 3KO hearts. (Troponin, Trop; Connexin-40, Cx40; Connexin-43, Cx43; Nuclear stain, DAPI). Scale bars: 50 µm (A–D), 100 µm (E,F).

The subset of 3KO embryos that reached later timepoints of development displayed marked overgrowth of the trabecular myocardium and ventricular conduction system. 3KO/CCS-LacZ hearts exhibited severe thickening of the His-Purkinje system (Fig. 8B,D,F). In the left ventricle of E17.5 3KO hearts, enlargement of the His bundle and left bundle branch resulted in LVOT obstruction (Fig. 8B). The right bundle branch and Purkinje fiber network were also hyperplastic and severely thickened (Fig. 8D). Disruption of the transmural cell cycle gradient in 3KO/CCS-LacZ hearts resulted in equilibration of nuclear densities within the VCS and the compact myocardial zones (Fig. 8F). To test whether aberrantly cycling VCS cells were appropriately lineage specified, we immunostained for Tbx3, Cx43, and Cx40 in E14.5 3KO hearts. The T-box repressor, Tbx3, is enriched in the proximal VCS (Fig. 9A), where it is known to negatively regulate the expression of Cx43 (Fig. 9C) (Hoogaars et al., 2004). Tbx3 expression was appropriately localized to the proximal VCS in 3KO hearts (Fig. 9B) with correspondingly lower levels of Cx43 in the His bundle (Fig. 9D). Tbx3 also regulates the expression of Cx40, which is maintained at lower levels within the proximal VCS compared to the remaining trabecular compartment (Fig. 9E,G,I). Cx40 low-expressing, left bundle branch cells (LBB) can be seen as a thin structure adjacent to the IVS (Fig. 9G) (Hoogaars et al., 2004). The remaining trabecular myocardium (Trab) expresses Cx40 at high levels (Fig. 9G,I). Although regional expression patterns of Cx40 were preserved in 3KO hearts (Fig. 9F,H,J), marked expansion of the His bundle and bundle branches significantly increased the proportion of Cx40 low-expressing trabecular myocytes within the ventricular chambers (Fig. 9H,J). The Cx40 high-expressing trabecular myocytes were marginalized to the lateral aspect of the LV chamber (Fig. 9H,J). The appropriate localization of VCS and trabecular markers suggests that transcriptional networks remain intact and functional despite loss of cell cycle control.

## Discussion

In this report, we explore the role of the pocket proteins in mediating VCS/trabecular myocyte cell cycle arrest. Based on *in vivo* cell cycle analysis using the FUCCI mouse system, the vast majority of VCS/trabecular myocytes arrest in G0/G1 upon specification. All three members of the pocket protein gene family are expressed within the trabecular region during development. Using a combinatorial knockout strategy, we demonstrate that p107/p130 dKO hearts show enhanced proliferation within zones of active myocyte cell cycling (compact myocardial zones and trabecular bases) resulting in delayed cardiac compaction and septal overgrowth *in utero* and biventricular outflow tract obstruction in the post-natal period. The remaining trabecular region and VCS cell cycle exit appropriately, thus preserving the transmural cell cycle gradient in dKO hearts. In contrast, cardiac 3KO null embryos manifest heart failure by E14.5–E15.5 from unrestrained trabecular myocyte cell cycling. Trabecular hyper-proliferation leads to complete disruption of the transmural cell cycle gradient and massive overgrowth of the VCS.

Formation of the ventricular conduction system has been well chronicled in the developing mouse heart (Virágh and Challice, 1977a; Virágh and Challice, 1977b; Virágh and Challice, 1980). Conduction myocytes develop within the trabecular region and can be distinguished from working myocardial cells by their glycogen enrichment and strong PAS staining (Viragh and Challice, 1977a; Virágh and Challice, 1977b). At E10.5, PAS^+^ cells of the primordial His-Purkinje system can be seen throughout the trabecular myocardium making direct interconnections with the AV nodal anlage (Virágh and Challice, 1977a; Virágh and Challice, 1977b). Early cell cycle arrest and maturation of the VCS and trabecular myocardium ensures direct coupling of the functional conduction system with the main force generating myocytes within the primitive heart.

The abundant expression of p130 in the trabecular myocardium is consistent with G0/G1 arrest in this region. P130 is known to function through the DREAM complex to maintain quiescence in mammalian cells in an Rb-independent manner (Litovchick et al., 2007). The DREAM complex, composed of DP, RB-like, E2F, and MuvB, binds to over 800 promoters in G0 and negatively regulates E2F transcriptional networks. P107 can compensate for p130 in this complex and maintain transcriptional repression of E2F targets (Litovchick et al., 2007). The ability of a single allele of p130 or p107 to maintain the transmural cell cycle gradient in the absence of Rb would support this model in murine heart development.

Defective heart and lung development in p107/p130 dKO mice expands the functional role of these genes during embryogenesis. Selective cell cycle perturbation within the compact myocardium and trabecular bases of dKO hearts suggests that the ability of Rb to compensate is limited within these regions. Whether this represents insufficient gene dosage of Rb or a cell type-specific dependency on p107 and p130 is uncertain. Despite regional cell cycling abnormalities in dKO hearts, the relative expression of trabecular markers remains normal, indicating that the ratio of compact to trabecular myocytes is unaffected. Therefore, the defect in dKO hearts is inappropriate timing of the switch between eccentric remodeling and concentric remodeling. The consequence of this developmental delay is abnormal elongation of the IVS and hyperplasia of the septal/parietal trabeculations resulting in biventricular outflow tract obstruction.

Unlike in the compact zone, Rb, p107, and p130 appear to function more redundantly in the trabecular myocardium. Disruption of the transmural cell cycle gradient requires the abolition of the entire pocket protein gene family. The potency of pocket proteins in the trabecular myocardium is likely due to the enrichment of Cip/Kip family CDK inhibitors, p21^CIP1^ and p57^Kip2^, in this region (Kochilas et al., 1999; Evans-Anderson et al., 2008). The Cip/Kip family members, p21^CIP1^, p27^Kip1^, and p57^Kip2^, modulate the activity of all G1/S CDK complexes, which in turn negatively regulate pocket protein function (Besson et al., 2008).

Although cardiac phenotypes have not been reported in p21^CIP1^ (Deng et al., 1995) or p57^Kip2^ (Zhang et al., 1997) individual or compound knockout mice, many phenotypic similarities are shared between pocket protein knockout mice and p21^CIP1^/p57^Kip2^ double knockout mice (Cobrinik et al., 1996; Zhang et al., 1999). p57 single knockout mice have defects in endochondral bone ossification of the long bones and the sternum (Zhang et al., 1997), and p21/p57 double knockout mice exhibit failure of lung alveolar development in the absence of cell cycling abnormalities (Zhang et al., 1999). All of these pathologic features are seen in p107/p130 dKO and 3KO mice in prior reports (Cobrinik et al., 1996) and newly reported here. The phenotypic similarities of p21/p57 double knockout mice to pocket protein dKO and 3KO mice are highly supportive of their molecular interactions *in vivo*. Further investigation of Cip/Kip knockout mice with an eye towards cardiac defects is therefore warranted. Furthermore, recent genome wide association studies have linked *CDKN1A* (p21) with QRS duration in European cohorts (Holm et al., 2010; Sotoodehnia et al., 2010). These studies are the first to link cell cycling genes with VCS function in the general population, and places new focus on pocket protein-dependent pathways as critical regulators of the specialized conduction system.

Proper expression and localization of VCS markers in 3KO hearts indicate that transcription factor networks remain intact despite loss of cell cycle control. CCS-LacZ, one of the earliest VCS markers, first appears in the ventricles at E9.5 and continues to be expressed in the specialized conduction system into adulthood (Rentschler et al., 2001). CCS-LacZ positive cells have limited proliferative potential in wildtype hearts, indicative of terminal differentiation. However, in 3KO mice VCS cells continue to proliferate and retain CCS-LacZ expression. In addition, Tbx3 is properly localized within the proximal VCS in 3KO hearts and continues to repress the expression of Cx43 and Cx40. These findings suggest that pocket protein expression and cell cycle exit are dispensible for cardiomyocyte specification towards a trabecular or conduction lineage. This would place specification and cell cycle regulation of VCS/trabecular myocytes in parallel rather than in series circuits. These data are in agreement with the work of Miquerol and Kelly who demonstrated that ventricular conduction cells are capable of limited rounds of cell division upon specification (Miquerol et al., 2010). Structural and functional maturation of the ventricular conduction system is known to continue well into the post-natal period, and the degree to which pocket protein deficiency affects patterning and ultimately function will need further investigation (Moskowitz et al., 2004).

Taken together, the pocket protein gene family functions as the principal regulator of VCS/trabecular myocyte cell cycle arrest. Graded loss of this gene family results in developmental abnormalities of the ventricular chambers and the cardiac conduction system.

## Materials and Methods

### Animal experimentation

All animal procedures were carried out in accordance with the Guide for the Care and Use of Laboratory Animals (U.S. National Institutes of Health Publication 86-23) and approved by the New York University School of Medicine Institutional Animal Care and Use Committee. Pocket protein mouse lines were a generous gift from Dr Lili Yamasaki. FUCCI constructs and mouse lines were purchased from AMALGAAM (MBL) (Niwa et al., 1991; Sakaue-Sawano et al., 2008).

### Western blot analysis

Western blot analysis was performed as previously described (Guo et al., 2009). Briefly, ventricles were harvested from embryos and adult hearts at indicated timepoints and cryopreserved in liquid nitrogen. Samples were then reconstituted in PBS, dounce homogenized, and then lysed with 1× SDS loading buffer. Protein concentration was determined using an RC DC kit (Bio-Rad), and equal amounts of protein were subjected to Western blot analysis. Proteins were detected with the following primary antibodies: mouse anti-human Rb (BD Pharmingen), mouse anti-p107 (SD9, Santa Cruz), rabbit anti-p107 (C-18, Santa Cruz), mouse anti-Rb2/p130 (BD Transduction Labs), anti-cyclin E (Upstate), rabbit anti-cyclin A (Abcam), rabbit anti-b2-microglobulin (Abcam). Bands were visualized by using the Odyssey Infrared Imaging System (LI-COR Biotechnology).

### Histology, immunofluorescence, and confocal analysis

Immunfluorescent and H&E staining were performed on formalin-fixed, paraffin-embedded samples and 4% PFA-fixed, cryosectioned samples, as previously described (Gutstein et al., 2001). Antibodies used were mouse anti-p107 (SD9, Santa Cruz), rabbit anti-p107 (C-18, Santa Cruz), mouse anti-Rb2/p130 (BD Transduction Labs), mouse anti-phospho-Histone H3 (Millipore), rabbit anti-Nkx2.5 (Abcam), anti-mouse Cx40 (Alpha Diagnostics Int), rabbit anti-Tbx3 (Abcam), rabbit anti-Cx43 (Sigma). β-galactosidase staining and whole mount visualization were performed as previously described (Rentschler et al., 2001). p107 and p130 positive nuclei were quantified in trabecular and compact myocardial regions as a percentage of total propidium iodide positive nuclei per region using Image J software (Collins, 2007). Quantification of pHH3 positive nuclei was performed per high power field (HPF) using comparable sections in mutant and control heart and lung regions.

### Laser capture microdissection (LCM)

LCM was performed as previously described (Trogan et al., 2002). Ventricular sections were harvested under RNAse-free conditions and embedded in OCT. Cyrosections were subsequently dried in graded ethanol solutions, followed by xylenes, and subsequently air-dried. Laser capture was performed under direct-microscopic visualization targeting trabecular versus compact myocardium onto thermoplast film caps (Arcturus Engineering). RNA was extracted using PicoPure RNA Isolation Kits (Arcturus Engineering) for quantitative RT-PCR analysis.

### Quantitative RT-PCR analysis

Total RNA was isolated from ventricular tissue at different developmental timepoints using PicoPure RNA Isolation Kits (Arcturus Engineering). Quantitative RT-PCR was performed using QuantiTect SYBR green RT-PCR Kit (Qiagen). The following primer sequences were used: see supplementary material Table S2.

### Echocardiography

Left ventricular dimensions and function were assessed by echocardiography as described previously with an ATL 5000CV Ultrasound System (Philips Medical, Bothell, WA) (Gutstein et al., 2001).

### Electrocardiography

Murine EKG was performed as previously described (Remo et al., 2011). Electrocardiogram analysis was performed using LabChart Pro EKG Analysis Software (ADInstruments).

### Electron microscopy

10 µm thickness frozen sections of mouse heart on the glass slides were fixed with 2.5% glutaraldehyde in 0.1M phosphate buffer (PB, pH 7.4) for 2 hours. After washing with PB, the samples were post fixed in 1% OsO_4_ for 1 hour, block staining with 1% uranyl acetate for 1 hour, dehydrate in ethanol and flat embedded in Araldite 502 (Electron Microscopy Sciences, Hatfield, PA). 60 nm En Face sections were cut, and stained with uranyl acetate and lead citrate by standard methods. Stained grids were examined under Philips CM-12 electron microscope (FEI; Eindhoven, The Netherlands) and photographed with a Gatan (4k ×2.7k) digital camera (Gatan, Inc., Pleasanton, CA).

### Statistical analysis

All statistical analyses were performed on Microsoft Excel using a 2-tailed, unpaired Student *t* test. A *P* value <0.05 was considered statistically significant.

## Supplementary Material

Supplementary Material
