# Supplementary material for: Pocket proteins critically regulate cell cycle exit of the trabecular myocardium and the ventricular conduction system
Source: Biol Open. 2013 Jul 31;2(9):968–78. doi: 10.1242/bio.20135785 (PMC3773344; doi:10.1242/bio.20135785)
Supplement: Supplementary Material [file supp_2_9_968__index.html]

Pocket proteins critically regulate cell cycle exit of the trabecular myocardium and the ventricular conduction system — Pocket proteins critically regulate cell cycle exit of the trabecular myocardium and the ventricular conduction system — Supplementary Material 

# Pocket proteins critically regulate cell cycle exit of the trabecular myocardium and the ventricular conduction system

## bio.20135785 Supplementary Material

**Files in this Data Supplement:**

- Supplementary Material - David S. Park et al. doi: 10.1242/bio.20135785
