# Supplementary material for: Pocket proteins critically regulate cell cycle exit of the trabecular myocardium and the ventricular conduction system
Source: Biol Open. 2013 Jul 31;2(9):968–78. doi: 10.1242/bio.20135785 (PMC3773344; doi:10.1242/bio.20135785)
Supplement: Supplementary Material [file supp_bio.20135785_bio.20135785-s1.pdf]

Biology Open

Supplementary Material

David S. Park et al. doi: 10.1242/bio.20135785

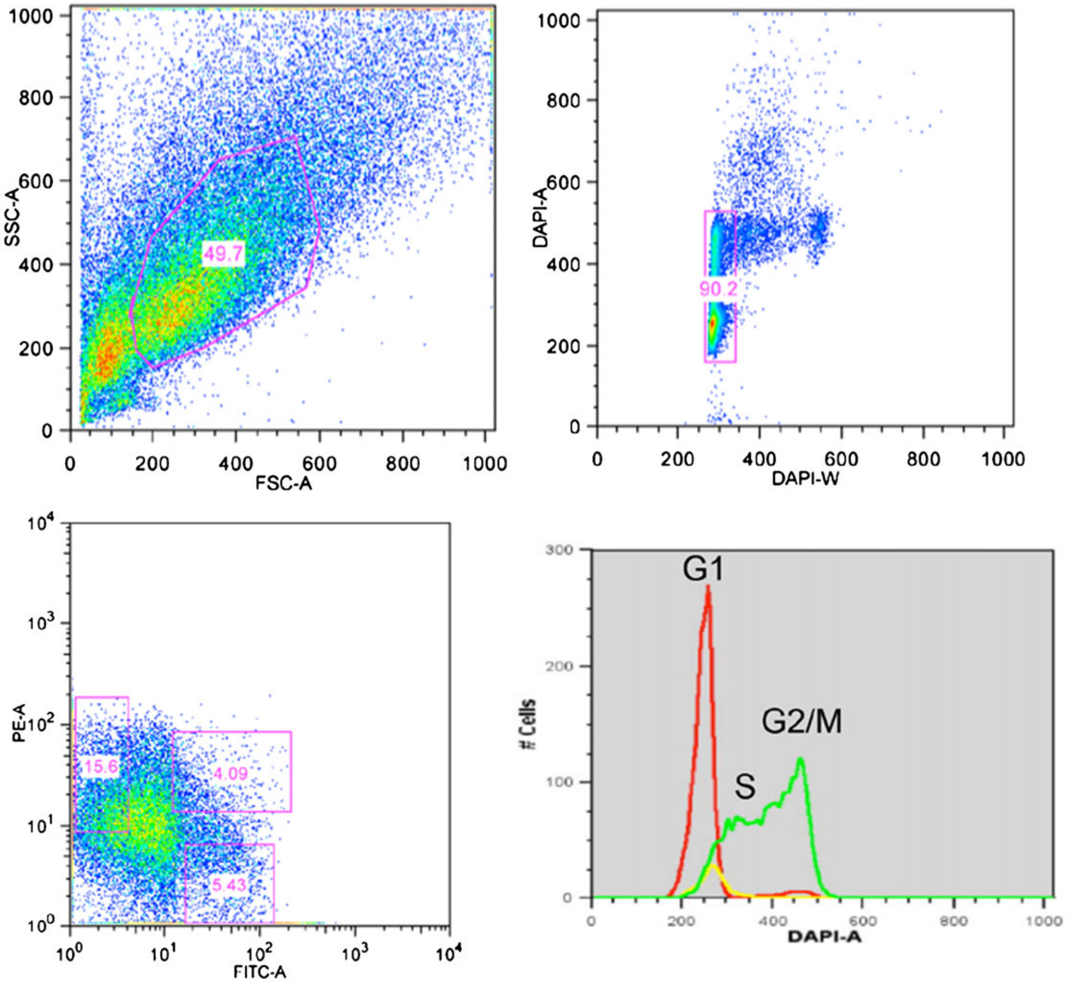

Fig. S1. DNA content frequency histograms of dissociated cardiomyocytes from FUCCI-Green and -Red double transgenic hearts at E12.5.

Biology Open

Table S1. EKG analysis of p107 and p130 single knockout mice compared to respective WT littermates (related to Fig. 3). No significant differences in EKG parameters were noted between the cohorts.

Genotype	Heart rate (BPM±S.D.)	PR interval (ms±S.D.)	P duration (ms±S.D.)	QRS interval (ms±S.D.)
p107 WT (n=6)	447±48	36.7±3.6	8.6±1.5	9.8±0.6
p107 KO (n=6)	400±71	38.6±3.8	9.1±0.9	10.2±0.7
p130 WT (n=6)	417±80	36.2±3.3	9.0±1.2	10.0±0.5
p130 KO (n=6)	450±46	39.3±2.1	8.7±1.2	10.6±0.7

Table S2. RT-PCR primer sequences.

Gene name	Primer sequence
Cdc25c forward	ATGTCTACAGGACCTATCCC
Cdc25c reverse	ACCTAAAACTGGGTGCTGAAAC
Cyclin A forward	CTCCTCCATGTCTGTGTTAA
Cyclin A reverse	CAACCTTACAGTTTGCAGGC
Rb forward	TGCATCTTTATCGCAGCAGTT
Rb reverse	GTTCACACGTCCGTTCTAATTTG
p16 forward	AAGGTGCCAGCCCCAATGTCCAAGATGC
p16 reverse	CCAAAAGGGGTGAGGAAAAACAAATGAG
p21 forward	TGTGGACATCACCCGTGACC
p21 reverse	GGAGAGGGCAGGCAGCGTAT
Cdk4 forward	AAGCCCGAGATCCCCACAGT
Cdk4 reverse	GGGCTCGGAAGGCAGAGATT
p107 forward	CAAGAGTCAAGGAAGTTCGCA
p107 reverse	TCGAAGGAGCAATTTTCAGCTTT
p130 forward	TCCTTACACGACGGTCTAGTG
p130 reverse	TCCCAGCGGGTAACACGTA
Nkx2-5 forward	GACAGGTACCGCTGTTGCTT
Nkx2-5 reverse	AGCCTACGGTGACCCTGAC
Cx40 forward	CCTGGATACCCTGCATGTCT
Cx40 reverse	GCTGTCGGATCTTCTTCCAG
Tbx5 forward	TGACTGGCCTTAATCCCAAA
Tbx5 reverse	ACAAGTTGTCGCATCCAGTG
Bmp10 forward	ACATCATCCGGAGCTTCAAGAACG
Bmp10 reverse	AACCGCAGTTCAGCCATGACG
E2F4 forward	CTCACCACCAAGTTCGTGTC
E2F4 reverse	TCTCGATCAGACCGATGCCTT
E2F5 forward	CCACCAAATTCGTGTCGTTGC
E2F5 reverse	AGCACCTACACCCTTCCACT
LacZ forward	ATAACGAGCTCCTGCACTGG
LacZ reverse	AAAAATCCATTTCGCTGGTG
S26 forward	GCCATCCATAGCAAGGTTGT
S26 reverse	GCCTCTTTACATGGGCTTTG
